# A study on identifying the phenotypic saturation thresholds of broomcorn millet based on functional limits and growth models

**DOI:** 10.1371/journal.pone.0334741

**Published:** 2025-11-07

**Authors:** Xiao Cui, Huiqin Li, Jiangchen Zan, Jianhua Cui, Pengzhi Hou, Linjuan Wang, Wenfeng Guo, Zhifang Bi, Fuzhong Li, Yuanhuai Han, Xiaoying Zhang, Yushen Wang

**Affiliations:** 1 School of Software, Shanxi Agricultural University, Jinzhong, China; 2 Department of Basic Sciences, Shanxi Agricultural University, Jinzhong, China; 3 College of Agriculture, Shanxi Agricultural University, Jinzhong, China; University of Florida Tropical Research and Education Center, UNITED STATES OF AMERICA

## Abstract

Broomcorn millet, renowned for its strong stress tolerance and rich nutritional value, serves as a crucial germplasm resource in the arid and semi-arid regions of northern China. It exhibits advantages such as a short growth period, high water use efficiency, salt tolerance, and pest resistance, which guarantee the stability of grain supply in local areas. Accurately identifying its growth saturation threshold is one of the core elements of precision agriculture technology and has become a research hotspot in the field of agricultural science both domestically and internationally in recent years.In this study, 8 representative broomcorn millet varieties were selected from typical dryland farming areas in Shanxi Province. Based on functional limits and growth models, temporal identification and comparative analysis of the phenotypic saturation thresholds of these 8 varieties were conducted, providing a scientific basis for variety selection and precision cultivation in arid regions.The Logistic model was used to fit the plant height growth dynamics, yielding a growth limit of 134.86–171.74 cm and a threshold achievement time of 59.60–73.80 days, with a model fitting degree R^2^ > 97%. The Richards model was applied to fit the stem diameter growth, resulting in a growth limit of 8.47–10.28 cm and a threshold achievement time of 70.50–182.20 days, with an R^2^ also > 97%. A quadratic polynomial regression model was employed to simulate the dynamic changes in chlorophyll content (R^2^ > 70%), clarifying the chlorophyll content characteristics of different plant parts.The results indicated significant differences in plant height, stem diameter, and chlorophyll content thresholds among different varieties. Pinshu 4 ranked first due to its dual advantages in plant height and stem diameter; Xiaohongruan Proso Millet 6 followed closely by virtue of its high photosynthetic efficiency; White Proso Millet 8 showed balanced performance in stem diameter and chlorophyll content; Jinshu 7 had stable plant height and relatively high chlorophyll content; the remaining varieties ranked lower due to weak performance in one or more traits.

## Introduction

Broomcorn millet, an ancient domesticated crop belonging to the Panicum in the Poaceae family, plays an irreplaceable ecological role in global agricultural ecosystems especially in arid and semiarid regions. Its unique stress tolerance to drought, salt-alkali [1], and barren soil resistance and high nutritional components (protein, dietary fiber, and phenolic compounds) make it a critical germplasm resource for food security and functional food development in dryland farming regions of northern China. With the development of genomics and phenomics technologies, the innovation of broomcorn millet germplasm resources has entered a high-throughput screening era, making it possible to the cultivation of new varieties [2,3] with high-yield potential and wide adaptation. However, the absence of variety-specific phenotypic threshold parameters remains a significant barrier to precision cultivation management and the full realization of varietal advantages.

In the field of crop growth modeling, multiscale model integration has emerged as an international research frontier. Systems such as the Agricultural Production Systems sIMulator (APSIM), developed by Jones, integrate biophysical processes with agronomic management modules, establishing a methodological frame work for dynamic feedback simulations of the crop-soil-atmosphere continuum [4]. These frameworks support yield risk assessments in major global grain-producing regions. Furthermore, plant morphological modeling based on fractal theory [5], research on Crop Growth Monitoring and Yield Estimation Based on Deep Learning Models [6], offering powerful tools for advancing precision agriculture. Practical studies on simplified cultivation techniques for intercropping broomcorn millet after spring wheat in the Ningxia Yellow River irrigation area, provide actionable insight for improving land use efficiency and agricultural productivity [7]. Xiaoning Cao [8] summarized cultivation practices, including sowing dates, planting density, fertilization strategies, and water-saving techniques, alongside process in understanding the stress physiology and photosynthetic mechanisms of broomcorn millet.

Despite these developments, research on threshold dynamics of phenotypic traits (e.g., plant height, stem diameter, and chlorophyll content) during the entire growth cycle of broomcorn millet remains limited. The distinct growth characteristics, environmental adaptability, and inter-varietal differences of broomcorn millet making it challenging to directly apply the methodologies developed for other crops. For instance, insufficient understanding of plant height thresholds may lead to suboptimal irrigation and fertilization timing, while neglecting stem diameter thresholds complicates the accurate evaluation of lodging resistance. Similarly, the lack of comprehensive research on chlorophyll content threshold impeded timely assessments of crop health.

This study aims to conduct field trials on eight different varieties of millet using the Logistic model, Richards model, and quadratic polynomial regression model to fit the growth changes in plant height, stem diameter, and chlorophyll content for each variety. Plant height reflects the growth potential of plants, stem diameter is associated with structural stability and nutrient transport, and chlorophyll content characterizes photosynthetic capacity and stress response. Clarifying these associations can make the selection of indicators more scientific and lay a foundation for the subsequent analysis of growth saturation thresholds.The study will test whether there are significant variety-specific differences in the saturation thresholds and the time required to reach these thresholds for plant height, stem diameter, and chlorophyll content among different millet varieties, and whether such differences can be precisely quantified by the models. This study will examine whether there is an intrinsic association between the threshold changes in plant height, stem diameter, and chlorophyll content, and whether the synchronous or asynchronous timing of reaching these thresholds reflects the degree of growth coordination among varieties. It will also investigate whether the selected models provide the optimal fit for each trait and whether the thresholds calculated using function limit theory can accurately characterize growth functional limits. It will verify whether there is asynchrony in the growth of plant height and stem diameter, and its association with variety stress tolerance, photosynthetic efficiency, and yield potential. By verifying these hypotheses, the phenotypic thresholds and threshold-reaching times of each variety will be obtained, differences between varieties will be compared and analyzed, and the correlations between indicators will be elucidated, thereby revealing the differences in growth dynamics among different varieties and filling the gap in research on phenotypic thresholds in millet. Systematically analyze the phenotypic saturation thresholds and threshold attainment times of eight varieties throughout their entire growth period, providing direct support for precision cultivation and the identification of variety advantages. The mathematical model’s threshold calculation method can also offer rapid and precise scientific methods for selecting high-quality germplasm resources in different regions, providing theoretical basis and data support for precision cultivation management, variety selection, and improvement of millet.

## Materials and methods

### Experimental materials

This experiment was conducted at the North Gate Experimental Field of Shanxi Agricultural University (37°25′N, 112°35′E), using broomcorn millet as the experimental material. Eight different varieties of millet were selected for cultivation, developed by the Key Laboratory of Loess Plateau Crop Genetic Resources and Germplasm Creation under the Ministry of Agriculture. These varieties exhibit a range of characteristics, including soft and hard textures, diverse colors, and representative genetic backgrounds. The names and sources of the experimental materials are shown in Table 1. By combining data model analysis, the study delved into the differences and characteristics of various millet varieties in terms of phenotypic traits.

**Table 1:** Name and source of test material.

Code	Variety name	Regions
1	Pinshu 1	Shanxi
2	Jinshu 5	Shanxi
3	Huangmizi3	Inner Mongolia
4	Pinshu4	Shanxi
5	Yuanpinghongmi5	Shanxi
6	Xiaohongruan Proso Millet 6	Ningxia
7	Jinshu 7	Shanxi
8	White Proso Millet 8	Inner Mongolia

**Fig 1:**
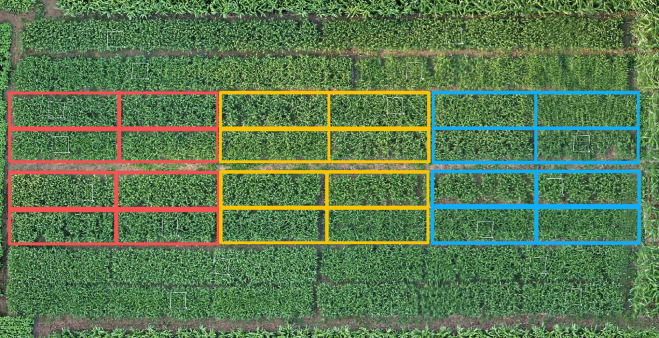
Field trial planting distribution.

All test materials were sown in early April 2022 and harvested at the end of September. The planting trial units were 2 m long and 0.7 m wide, with an area of 1.4 m2 each. The row spacing was 30 cm, and there were 24 trial units in total. Three to five seeds were sown per hole. In the field, each sample was replicated three times, with each replicate planted with eight varieties. Ten plants were sampled from each variety, totaling 30 plants across the three replicates, with the average value representing the phenotypic trait level of the tested variety. The field trial planting distribution is shown in Fig 1.

### Data collection

**Fig 2:**
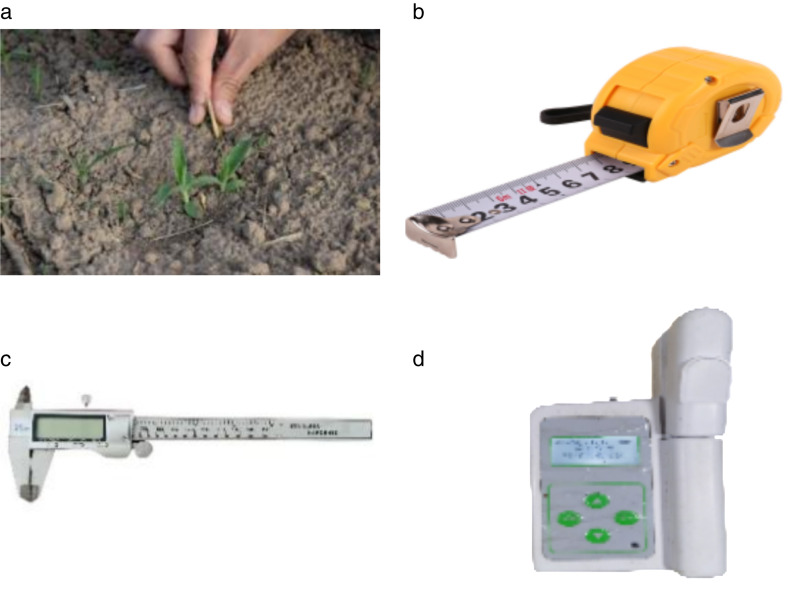
The field sampling site and test samples and tools. (a) presents the field sampling points and test samples; (b) is a tape rule; (c) is a dial caliper; (d) is a chlorophyll meter.

Phenotypic data were collected from June 5 to August 21, 2022, covering key growth stages: seedling, jointing, heading, grain filling, and maturity. Plant height was measured as the vertical distance from the coleoptile node to the tip of the longest leaf using a ruler (range: 100 cm; accuracy: ± 0.1 cm). Stem diameter was measured at the first and second nodes above the root base using a caliper (range: 150 mm; accuracy: ± 0.01 mm), with the average of maximum and minimum diameters recorded [9]. Chlorophyll content was quantified using a portable chlorophyll meter, with measurements taken at the base, middle, and top of five leaves per plant. The mean values were used for further analysis [10]. The field sampling site, test samples, and tools are shown in Fig 2. The items and standards for morphological identification of broomcorn millet are shown in Table 2.

**Table 2:** Projects and standards for morphological identification of broomcorn millet.

Indicator	Standard
Plant height	The length from the base of the stem to the highest point. Survey 30 plants and take the average value. Unit: cm
Stem diameter	lace the caliper around the stem, adjust the screw until the two jaws are tightly against the stem, and read the data
Chlorophyll	Select the flag leaf, avoid the main vein and coarse veins when measuring, and measure three times at the upper, middle, and lower parts of the leaf

### Data analysis

The phenotypic data of plant height, stem thickness, and chlorophyll content of the eight varieties were collected and integrated using excel 2010. To ensure data integrity, all the data at each growth stage were scrutinized for missing or anomalous entries [11]:

The R language is used to detect and handle the missing values in the data based on the characteristics of the data. If the number of missing values is small and randomly distributed, the corresponding records were removed to minimize uncertainty [12]; Conversely, if the missing values have a certain pattern or a substantial volume, the interpolation method was used. A common method is that using the mean value of the variable to replace the missing values, or regression model-based interpolation, where other relevant variables are used to predict missing values.

Outliers were identified and removed using the triple standard deviation method. Firstly, a scatter plot was generated using the R language on the raw data to identify outliers that significantly deviate from the normal data range. The cause of these outliers was thoroughly investigated, which could induce by measurement errors such as malfunction of measuring instruments and human recording errors, and such outliers were removed directly. However, if the outliers were attributed to unique biological phenomena—such as unusual growth responses due to specific environmental stimuli, pest infestations, or diseases—they were retained based on their biological significance. And these outliers were individually analyzed in the subsequent analysis, with appropriate adjustments to make them more consistent with the overall data trends.

The pre-processed data were analyzed using Origin2019 to create scatter plots, label the fitted curves and visualize the results. The logistic growth model was used to fit the plant height values of each variety, while Richards’ model was used to fit the plant diameter values of each variety, and polynomial regression was used to analyze the chlorophyll content of each variety. The effectiveness of each model was evaluated using the coefficient of determination R^2^, where a higher coefficient indicated a better fit of model to the data [13–15].

### Experimental methods

The Crop Growth Simulation Model (CGSM) is an effective tool for studying the growth patterns of crops. Li et al. used wheat and fava beans as research subjects, employing crop growth dynamic data from a wheat-fava bean intercropping system and the Logistic growth model to conduct their study, thereby providing scientific basis for understanding the interactions between crops in both monocropping and intercropping systems. Using mathematical formulas to express the changes in various crop measurement indicators can help uncover the underlying patterns of crop phenotypes. Growth rate is a parameter that describes the speed at which crop phenotypic traits increase over a unit of time, reflecting the growth intensity of crops at specific growth stages. The plant height growth rate parameter in this study reflects the rate of growth as plant height approaches its growth limit; the larger the value, the faster the growth of plant height at the corresponding stage. The stem diameter growth rate parameter characterizes the expansion speed of stem diameter over time, with its magnitude directly reflecting the activity level of radial stem growth. The shape parameter is a parameter describing the morphological characteristics of the growth model fitting curve, determining the overall contour and trend of the curve. In the Richards model, shape parameters influence the curvature of the stem diameter growth curve and the position of inflection points. Different shape parameter values correspond to distinct growth dynamic patterns, enabling differentiation of morphological differences in stem diameter growth among different millet varieties. Based on this, this study selected eight different varieties, including Pinshu 1, Jinshu 5, and Huangmizi 3, for field planting experiments. The aim was to conduct in-depth research on the threshold changes in millet phenotypic traits through data modeling, providing a solid theoretical basis for precise cultivation management and variety selection of millet, and promoting the high-quality development of the millet industry.

This study selected eight different varieties for field planting. The eight varieties tested were: millet variety Pinshu 1, Jinshu 5, Huangmizi 3, Pinshu 4, Yuanpinghongmi 5, Xiaohongruan Proso Millet 6, Jinshu 7, and White Proso Millet 8. For convenience of notation, denotes the plant height of the eight millet varieties, represents the stem diameter of the eight millet varieties, and represents the chlorophyll content of the eight millet varieties. This experiment aims to reveal the differences in growth dynamics among different millet varieties by studying the threshold changes in phenotypic traits (plant height, stem diameter, and chlorophyll content) throughout the entire growth period. Specifically, based on the Logistic model, Richards model, and quadratic polynomial regression model, we conducted fitting analyses on the plant height, stem diameter, and chlorophyll content of different varieties, obtained the thresholds for these phenotypic traits and the time required to reach these thresholds for each variety, and compared the time required for each variety to reach its mature stable height. Additionally, the correlations among the three phenotypic traits were analyzed to provide data support for millet production and variety improvement.

## Analysis of change in plant height thresholds of eight varieties based on Logistic model

### Establishing plant height growth model based on Logistic theory

Logistic growth model, proposed by mathematical biologist Pierre-Francois Verhulst in the middle of the 19th century, serves as an extension of the Malthusian population model [16]. This nonlinear growth model is wide applicable, its discrete point columns are monotonically increasing bounded point columns, and the limits represent the crop growth and maturity reached a steady state [17]. The simulation model is as follows Eq (1):


$$Height(i)=\frac{L}{1+e^{-k*(t-t_{0})}},i=1,2,\cdots ,8.$$ (1)

where Height(i) represents the response variable of plant height; L represents the maximum plant height value at the saturation threshold of plant growth; K denotes the rate of plant rise; $t_{0}$ denotes the time when the plant reaches half of its maximum growth limitation; t represents the time variable.

**Fig 3:**
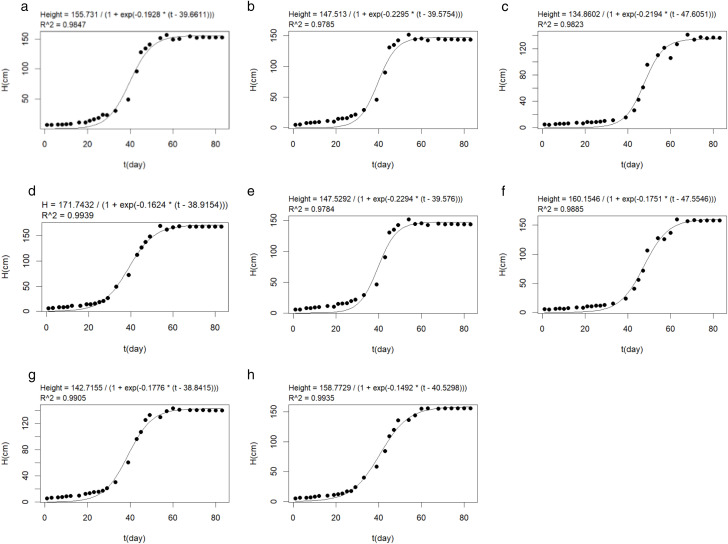
Logistic model fitting curves of plant height in eight varieties. (a) represents the fitting curve of the plant height of Pinshu 1; (b) represents the fitting curve of the plant height of Jinshu 5; (c) represents the fitting curve of the plant height of Huangmizi 3; (d) represents the fitting curve of the plant height of Pinshu 4; (e) represents the fitting curve of the plant height of Yuanpinghongmi 5; (f) represents the fitting curve of the plant height of Xiaohongruan Proso Millet 6; (g) represents the fitting curve of the plant height of Jinshu 7; (h) represents the fitting curve of the plant height of White Proso Millet 8.

In this experiment, the plant height of eight varieties of broomcorn millets were analyzed with a logistic model and the simulation effect was determined. The fitted parameters of different varieties are summarized in Table 3, and the corresponding fitting results are shown in Fig 3.

**Table 3:** Logistic Model Parameters of Plant Height in Different Broomcorn Millet.

Variety	Growth Limit L (cm)	Growth Rate k	Time to Reach Half of the Upper Limit Value $t_{0}$ (days)	$R^{2}$
Pinshu 1	155.731	0.1928	39.6611	0.9847
Jinshu 5	147.513	0.2295	39.5754	0.9785
Huangmizi3	134.8602	0.2194	47.6051	0.9823
Pinshu4	171.7432	0.1624	38.9154	0.9939
Yuanpinghongmi5	147.5292	0.2294	39.576	0.9784
Xiaohongruan Proso Millet 6	160.1546	0.1751	47.5546	0.9885
Jinshu 7	142.7155	0.1776	38.8415	0.9905
White Proso Millet 8	158.7729	0.1492	40.5298	0.9935

### Analysis of time threshold for the Plant Height based on the definition scale of functional limit

According to the Monotone Convergence Theorem, as t→∞, the limit of the Logistic model Eq (2) approaches the asymptotic value L, i.e.,


$$\underset{t\to \infty }{\lim}Height(i)=\underset{t\to \infty }{\lim}\frac{L}{1+e^{-k*(t-t_{0})}}=L,i=1,2,\cdots ,8.$$ (2)

Expressed in the ε−T formalism, this means: for any ∀ε>0, there exists T>0 such that when t>T in Eq (3).


$$\left|\frac{L}{1+e^{-k*(t-t_{0})}}-L\right|<\varepsilon .$$ (3)

According to the definition of functional limits, T is not unique. Thereby, by solving for the first occurrence $T_{ZG}$, the moment when the crop height reaches the asymptotic value L, defines the threshold time for growth cessation. Beyond $T_{ZG}$, plant height growth nearly stops, marking the onset of maturity initiation, during which kernels begin to fill out.

Since depends on the scale ε, we calculate the time thresholds $T_{ZG}$ for eight crop varieties under ε=0.01. The results are summarized in Table 4.

**Table 4:** Approximate Threshold Values of Plant Height in Different Broomcorn Millet.

Variety	Height(i)	ε values	L (cm)	$T_{ZG}$ (day)	$R^{2}$
Pinshu 1	Height(1)	0.01	155.731	63.4947	0.9847
Jinshu 5	Height(2)	0.01	147.513	59.5977	0.9785
Huangmizi3	Height(3)	0.01	134.8602	68.5491	0.9823
Pinshu4	Height(4)	0.01	171.7432	67.2104	0.9939
Yuanpinghongmi5	Height(5)	0.01	147.5292	59.6070	0.9784
Xiaohongruan Proso Millet 6	Height(6)	0.01	160.1546	73.7974	0.9885
Jinshu 7	Height(7)	0.01	142.7155	64.7149	0.9905
White Proso Millet 8	Height(8)	0.01	158.7729	71.3281	0.9935

According to Table 4, under the same ε -value condition (ε=0.01), the ideal plant height among different broomcorn millet varieties were significantly different, and the required time to reach the ideal plant height also varied, notably, the ideal plant height of Pinshu4 was the highest, while that of Huangmizi3 was the lowest. Although there was significant difference between the plant height of these two varieties, the time to reach the ideal plant height was almost the same, which were 67.2104 days for Pinshu4 and 68.5491 days for Huangmizi3 respectively. It suggests that there was no significant correlation between ideal plant height and crop maturation rate.

Additionally, the results indicated that the time to reach the ideal plant height of Jinshu5 and YuanpingHongmi5 is the shortest, while Xiaohongruan Proso Millet 6 and White Proso Millet 8 needed more time to reach the ideal plant height. These findings indicated that the growth dynamics shown significant difference among different varieties of broomcorn millet.

The differences in plant height among varieties may be closely linked to multiple factors, such as genetic background, growth environmental conditions, and cultivation management practices.

### Variance analysis and mean analysis of plant height

This experiment plotted box plots and mean plots for the plant height data of eight millet varieties and performed analysis of variance (ANOVA). The box plots of plant height show the distribution characteristics, dispersion, and outliers of the data, while the mean plots display the central tendency of the plant height data. Together, they comprehensively reflect the differences between varieties. The p -value is used to determine whether there is a significant effect of variety on plant height. A smaller p -value indicates a more significant difference in plant height between varieties, while a larger p -value indicates a less significant difference.

**Fig 4:**
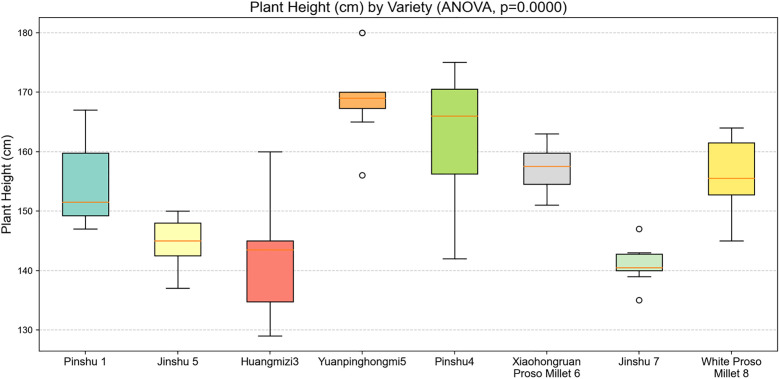
Box plot of plant height of different varieties of millet.

As shown in Fig 4, the box plots of plant height indicate significant differences in plant height among different varieties of millet (ANOVA, p < 0.0001). From the box plots, it can be observed that the Pinshu4 variety has a generally higher plant height, with the upper boundary of the box approaching 180 cm, and there are outliers at high values; while the Jinshu 7 variety has a notably lower plant height, with the box plot concentrated around 140 cm, reflecting its genetic or environmental adaptability leading to a shorter plant height. The significant differences in the median plant height between varieties indicate that breeding millet varieties with different plant height characteristics has a phenotypic basis.

**Fig 5:**
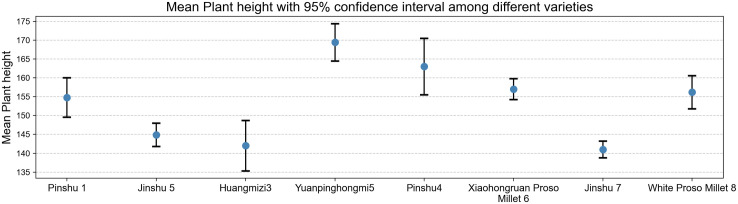
Average plant height of different varieties of millet.

As shown in Fig 5, the mean plant height data indicate that the Yuanpinghongmi5 and Pinshu4 varieties exhibit a significant height advantage, with means approaching or exceeding 170 cm, and longer confidence intervals, reflecting rich genetic diversity in plant height traits and potentially harboring superior genes for tall plant types; The mean plant height of the Jinshu 7 variety was significantly lower than that of other varieties, with a short confidence interval, indicating that its plant height trait is relatively stable and tends toward dwarfism, which may have potential value for breeding dwarf, lodging-resistant varieties. The differences in plant height among varieties provide diverse germplasm options for plant architecture breeding.

## Analysis of threshold change of stem diameter based on the Richards model

### Development of a stem diameter growth model based on Richards’ theory

The Richards model, proposed by American civil engineer L. A. Richards in 1931 was developed as a mathematical framework to describe the movement of water in soil [18]. Rooted in the principles of mass conservation and hysteresis effects, this model simulates the process of water absorption from soil into plant roots. As a nonlinear growth model, The Richards model serves as a foundational framework from which other growth equations, such as the Mitscherlich equation, Gompertz equation, and Logistic equation, can be derived. Due to its flexibility and accuracy, it has been widely adopted in plant growth studies [19] and is particularly effective in simulating temporal changes in crop stem diameter. The model is mathematically represented as Eq (4):


$$ZJ(i)=A(1-e^{-kt}{)}^{n},i=1,2,\cdots ,8.$$ (4)

Where ZJ(i) represents the response variable of stem diameter, A represents the asymptotic limit of crop growth, K denotes the crop growth rate, n denotes the shape parameter which determines the curve’s shape, and t is the time variable.

**Fig 6:**
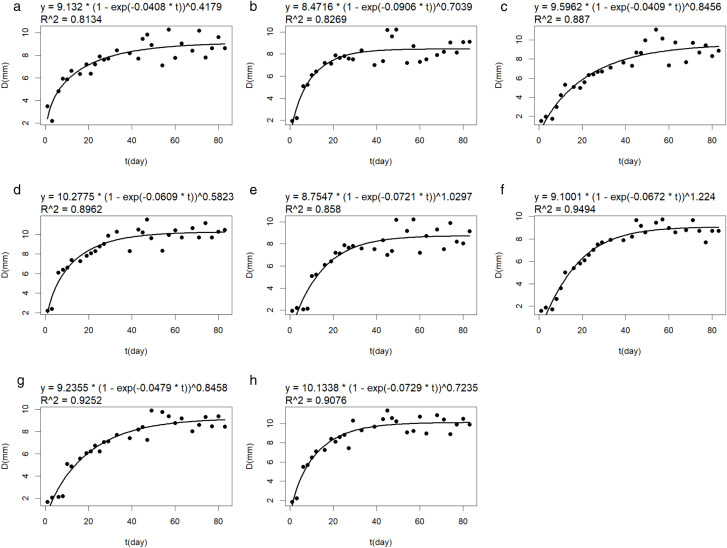
Richards model fitting curves of stem diameter  for eight varieties. (a) shows the fitting curve of the stem diameter of Pinshu 1; (b) shows the fitting curve of the stem diameter of Jinshu 5; (c) shows the fitting curve of the stem diameter of Huangmizi3; (d) shows the fitting curve of the stem diameter of Pinshu4; (e) shows the fitting curve of the stem diameter of Yuanpinghongmi5; (f) shows the fitting curve of the stem diameter of Xiaohongruan Proso Millet 6; (g) shows the fitting curve of the stem diameter of Jinshu 7; (h) shows the fitting curve of the stem diameter of White Proso Millet 8.

In this experiment, the Richards model was used to fit the stem diameter of eight varieties of broomcorn millet. The simulation effectiveness was determined by $R^{2}$. Table 5 presents the parameters obtained from fitting different varieties, while Fig 6 illustrates the fitting results.

**Table 5:** Comparison of Richards Model Parameters for Stem Diameter of Different Proso Millet Varieties.

Variety	Asymptotic Limit A (cm)	Growth Rate k	Shape Parameter n	$R^{2}$
Pinshu 1	9.132	0.0408	0.4179	0.8134
Jinshu 5	8.4716	0.0906	0.7039	0.8269
Huangmizi3	9.5962	0.0409	0.8456	0.887
Pinshu4	10.2775	0.0609	0.5823	0.8962
Yuanpinghongmi5	8.7547	0.0721	1.0297	0.858
Xiaohongruan Proso Millet 6	9.1001	0.0672	1.224	0.9494
Jinshu 7	9.2355	0.0479	0.8458	0.9252
White Proso Millet 8	10.1338	0.0729	0.7235	0.9076

### Analysis of threshold for the stem diameter growth based on the definition scale of functional limit

According to the Monotone Convergence Theorem, when t→∞ the limit of the Richards model approaches the asymptotic value A, that is Eq (5),


$$\frac{\lim}{t\to \infty }ZJ(i)=A(1-e^{-kt}{)}^{n}=A,i=1,2,\cdots ,8.$$ (5)

Furthermore, it can be expressed in the ε−T language, that is ∀ε>0, ∃T>0, such that when t>T in Eq (6).


$$\left|A{\left(1-e^{-kt}\right)}^{n}-A\right|<\varepsilon .$$ (6)

According to the definition of a function’s limit, the parameter T is not uniquely determined. Consequently, the time $T_{ZJ}$ at which the limit value A is first reached represents the threshold moment when the crop’s stem diameter growth reached its limit. Beyond this point, the growth of the stem diameter nearly ceases, marking the onset of early maturity, and the stem approaches its maximum thickness [20].

Since the calculation of T depends on the scale ε, given ε=0.01, the threshold $T_{ZJ}$ of eight varieties reaching the ideal stem diameter value A was determined. By substituting the value of $T_{ZJ}$ calculated at ε=0.01 into the Eq (6), the stem diameter value $y_{ZJ}$ to the first attainment of the ideal plant height at this scale was computed. Detailed results were presented in Table 6.

**Table 6:** Stem Diameter Thresholds of Different broomcorn Millet Varieties.

Variety	ZJ(i)	ε	A /(cm)	$T_{ZJ}$ (day)	Stem Diameter Value$y_{ZJ}$ /mm	$T_{ZJ}$ (day)	$R^{2}$
Pinshu 1	ZJ(1)	0.01	9.132	63.4947	7.0709	145.78	0.8134
Jinshu5	ZJ(2)	0.01	8.4716	59.5977	8.4446	70.50	0.8269
Huangmizi3	ZJ(3)	0.01	9.5962	68.5491	9.1022	182.20	0.887
Pinshu4	ZJ(4)	0.01	10.2775	67.2104	10.1773	104.79	0.8962
Yuanpinghongmi 5	ZJ(5)	0.01	8.7547	59.6070	8.6321	94.29	0.858
Xiaohongruanmi 6	ZJ(6)	0.01	9.1001	73.7974	9.0220	102.79	0.9494
Jinshu7	ZJ(7)	0.01	9.2355	64.7149	8.8823	139.01	0.9252
Whitemizi 8	ZJ(8)	0.01	10.1338	71.3281	10.0933	90.23	0.9076

Research revealed significant differences in the maximum stem diameter among different varieties. Each variety displayed distinct maximum plant heights and thickest stem diameters, with Pinshu4 exhibiting the highest values for both plant height and stem diameter. Further analyzing showed that when ε is set to 0.01, the time at which the plant height reached its peak (as calculated by the plant height formula) was substituted into the stem diameter formula to determine the stem diameter of the variety at that specific time point. A comparison between the maximum stem diameter (A) and the stem diameter ($y_{ZJ}$) calculated at the time of maximum plant height indicated that stem diameter reached its peak after the plant height essentially reached its maximum. Differences between the maximum stem diameter and the stem diameter at the time of maximum plant height were observed across all eight varieties, with the most remarkable difference was observed in Pinshu1. Data in Table 6 highlighted the discrepancies, and the corresponding figures also visually confirmed that stem diameter continues to grow at a relatively fast rate even when plant height has reached its maximum.

Meanwhile, the significant difference was also observed between T and $T_{ZJ}$, which indicated that when plant height reached the expected ideal height, the stem diameter has not yet achieved the ideal size. This phenomenon indicated an asynchrony between radial and longitudinal growth. The rate of longitudinal elongation is significantly faster than that of radial expansion during stem morphogenesis. This allometric growth characteristic may be due to the assimilate partitioning patterns [21] or endogenous hormones [22,23]. The stem diameter threshold $T_{ZJ}$ represents a critical turning point in the growth of crop stem diameter. At this stage, the growth rate of stem diameter significantly decreases, signaling the onset of the early maturation phase.

### Variance analysis and mean analysis of stem diameter

Box plots and mean plots clearly show the stem diameter characteristics of eight different varieties of millet, enabling a comprehensive understanding of the overall distribution characteristics and variability of stem diameter among different varieties, as well as an accurate grasp of differences in their average levels. This provides intuitive and reliable data support for breeding superior varieties with stem diameters that meet breeding objectives such as resistance to lodging and high light efficiency, thereby enhancing the targeting and efficiency of variety selection.

**Fig 7:**
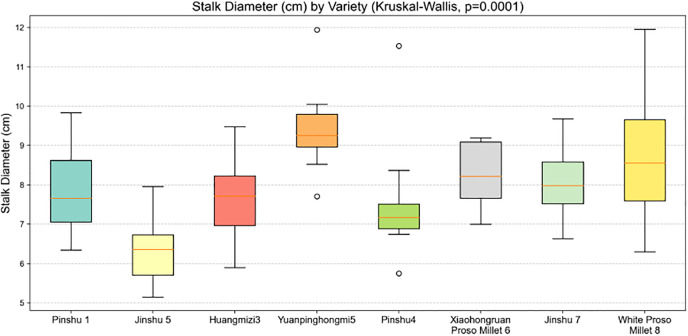
Box plot of millet stems of different varieties.

As shown in Fig 7, based on the Kruskal-Wallis test (p = 0.0001), there are significant differences in stem thickness among varieties. The White Proso Millet 8 variety exhibits outstanding stem thickness, with box plots and whisker charts extending to 12 cm, a high median, and large data dispersion, potentially indicating stronger mechanical support potential; The Jinshu 5 variety has relatively thin stems, with the box plot concentrated in the 5–8 cm range, indicating that stem thickness traits show significant differentiation among varieties, and germplasm resources can be selected based on stem thickness requirements.

**Fig 8:**
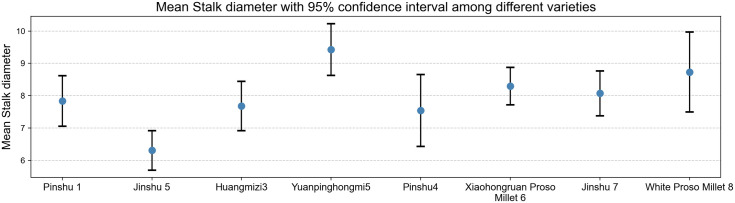
Average stem height of different varieties of millet.

As shown in Fig 8, the mean stem thickness and confidence intervals reflect significant differences in stem development among varieties. The Yuanpinghongmi5 variety has the highest mean stem diameter and a wide confidence interval, indicating robust stems with high genetic variability, performing exceptionally well in agronomic traits such as lodging resistance and mechanical support; the Jinshu 5 variety has a low mean stem diameter and a narrow confidence interval, with relatively slender stems and stable traits. This differentiation in stem thickness among varieties provides phenotypic evidence for selecting suitable varieties for dense planting and lodging resistance, and can be combined with yield and other traits for targeted breeding.

## Threshold variation analysis of chlorophyll content in eight proso millet varieties based on polynomial models

### Construction of chlorophyll model based on polynomials

The polynomial model is a widely utilized fitting tool that effectively captures morphological variation characteristics of crops across different growth stages [24]. By adjusting the polynomial order and coefficients, this model can more precisely describe the variation patterns of crops during distinct growth phases, thereby improving the understanding of crop growth dynamics and physiological changes. The simulation model is expressed as follows Eq (7):


$$YL(i)=at^{2}+bt+c,i=1,2,\cdots ,8.$$ (7)

**Fig 9:**
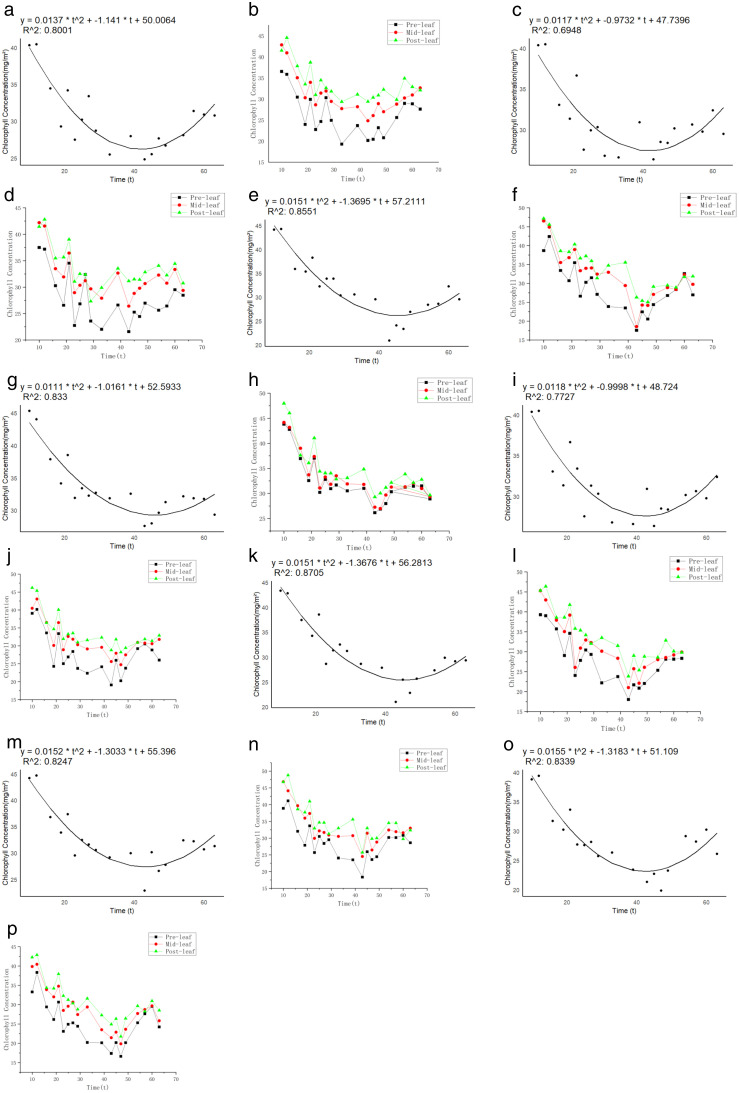
Fitting curves of chlorophyll content for eight varieties. (a) shows the chlorophyll content fitting curve of Pinshu 1; (b) shows the chlorophyll content line chart of the basal, middle, and tip parts of Pinshu 1 leaf; (c) shows the chlorophyll content fitting curve of Jinshu 5; (d) shows the chlorophyll content line chart of the basal, middle, and tip parts of Jinshu 5 leaf; (e) shows the chlorophyll content fitting curve of Huangmizi3; (f) shows the chlorophyll content line chart of the basal, middle, and tip parts of Huangmizi3 leaf; (g) shows the chlorophyll content fitting curve of Pinshu4; (h) shows the chlorophyll content line chart of the basal, middle, and tip parts of Pinshu4 leaf; (i) shows the chlorophyll content fitting curve of Yuanpinghongmi5; (j) shows the chlorophyll content line chart of the basal, middle, and tip parts of Yuanpinghongmi5 leaf; (k) shows the chlorophyll content fitting curve of Xiaohongruan Proso Millet 6; (l) shows the chlorophyll content line chart of the basal, middle, and tip parts of Xiaohongruan Proso Millet 6 leaf; (m) shows the chlorophyll content fitting curve of Jinshu 7; (n) shows the chlorophyll content line chart of the basal, middle, and tip parts of Jinshu 7 leaf; (o) shows the chlorophyll content fitting curve of White Proso Millet 8; (p) shows the chlorophyll content line chart of the basal, middle, and tip parts of White Proso Millet 8, it can be inferred that the chlorophyll content of most varieties followed a “unimodal curve”, with peaks concentrated during the heading to grain-filling stages (e.g., Pinshu1 and Huangmizi3).Chlorophyll, being fundamental to photosynthesis, suggested that this stage likely represented the period of maximum photosynthetic activity in broomcorn millet, providing essential energy for grain filling.

In this study, the chlorophyll content of eight different varieties of broomcorn millet was analyzed using the polynomial model (7), and the fitting performance was determined by $R^{2}$. Data in Table 7 showed the fitted parameters of each variety, along with the corresponding chlorophyll content values when the broomcorn millet reached the limit $YL_{T_{ZG}}$ of plant height. The left portion of Fig 9 represented the overall variation curves of chlorophyll content across different varieties, while the right portion of Fig 9 depicted the change of Chlorophyll content for the base of leaf (BL), middle of leaf (MF), and tip of leaf (TL) over time, represented as polyline graphs.

**Table 7:** Parameters of chlorophyll content models for different broomcorn millet varieties.

Variety	Fitting Model	$T_{ZG}$ (day)	$YL_{T_{ZG}}$	$R^{2}$
Pinshu 1	$YL(1)=0.0137\cdot t^{2}-1.141\cdot t+50.0064$	63.4947	32.7420	0.8001
Jinshu 5	$YL(2)=0.0117\cdot t^{2}-0.9732\cdot t+47.7396$	59.5977	31.2514	0.6948
Huangmizi3A	$YL(3)=0.0151\cdot t^{2}-1.3695\cdot t+57.2111$	68.5491	34.2871	0.8551
Pinshu4A	$YL(4)=0.0111\cdot t^{2}-1.0161\cdot t+52.5933$	67.2104	34.4877	0.833
Yuanpinghongmi5	$YL(5)=0.0118\cdot t^{2}-0.9998\cdot t+48.724$	59.6070	31.1698	0.7727
Xiaohongruanmi 6	$YL(6)=0.0151\cdot t^{2}-1.3676\cdot t+56.2813$	73.7974	37.4013	0.8705
Jinshu 7	$YL(7)=0.0152\cdot t^{2}-1.3033\cdot t+55.396$	64.7149	34.6980	0.8247
Whitemizi 8	$YL(8)=0.0155\cdot t^{2}-1.3183\cdot t+51.109$	71.3281	36.1363	0.8339

### Analysis of chlorophyll content threshold based on polynomial model

Combined with the data presented in Fig 9.

When plant height first reaches its ideal maximum, the plant entered a critical phase of rapid growth. The chlorophyll content at this time reflects the photosynthetic capacity of the variety during its growth peak. Xiaohongruan Proso Millet 6 and White Proso Millet 8 exhibit higher chlorophyll content at the critical time point, which typically correlates with stronger photosynthetic activity, thereby promoting biomass accumulation and yield improvement [25].

The chlorophyll content fitting curve of Jinshu5 showed a lower initial value and faster decline, likely due to its earlier transition into maturity. This raises the concern about the risk of premature senescence impacting yield [26]. The chlorophyll distribution characteristics in different regions of leaf (base, middle, and tip) in Fig 9 revealed notable variations within the same variety. Understanding chlorophyll content at different leaf positions allows for a more comprehensive assessment of crop growth and development. Monitoring chlorophyll content at the leaf tip can provide earlier indicators of insufficient light, nutrient deficiency, or pest stress, allowing timely adjustments in planting density, fertilization, or irrigation strategies. Xiaohongruan Proso Millet 6 displays minimal differences in chlorophyll content across leaf positions, indicating uniform physiological leaf status and strong stress resistance, making it suitable for promotion in regions with significant environmental fluctuations [27].

### Chlorophyll variance analysis and mean analysis

Box plots and mean plots clearly show the characteristics of chlorophyll content in eight different varieties of millet, reflecting the overall distribution characteristics and variability of chlorophyll content in each variety, accurately grasping the differences in average levels, and providing intuitive and reliable data support for selecting superior varieties with chlorophyll content that meets breeding objectives such as photosynthetic efficiency and stress tolerance.

**Fig 10:**
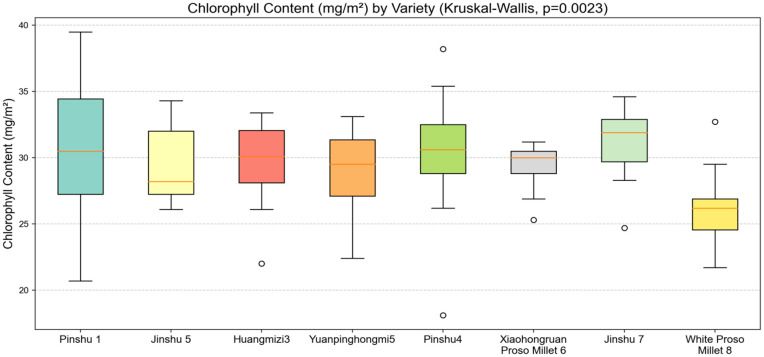
Box  plot of chlorophyll content in different varieties of millet leaves.

As shown in Fig 10, the box plot analysis of chlorophyll content revealed significant differences among varieties following the Kruskal-Wallis test (p = 0.0023). The Pinshu 1 variety exhibited a clear advantage in chlorophyll content, with box plots concentrated between 30 and 40 mg/m^2^, indicating strong photosynthetic pigment accumulation capacity and potentially higher photosynthetic efficiency; The White Proso Millet 8 variety has relatively low chlorophyll content, with the box and whisker plot showing data concentrated between 20 and 30 mg/m^2^, reflecting the differentiation of photosynthetic physiological characteristics among different varieties. This can be combined with yield traits to screen for high-photosynthetic-efficiency varieties.

**Fig 11:**
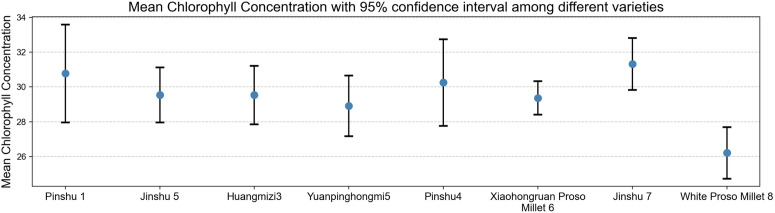
Average chlorophyll content of different varieties of millet leaves.

As shown in Fig 11, the mean chlorophyll concentrations and 95% confidence intervals of different proso millet varieties exhibit significant differences. The mean chlorophyll concentrations of the Pinshu 1 and Jinshu 7 varieties are relatively high, with broader confidence intervals, indicating a larger range of chlorophyll content variability and potentially rich genetic variation in photosynthetic potential; In contrast, the White Proso Millet 8 variety had a significantly lower mean value and a narrower confidence interval, suggesting that this variety has poorer stability in chlorophyll synthesis or accumulation, and is distinctly differentiated from other varieties in terms of photosynthetic physiological characteristics, making it an important reference for high light-use efficiency breeding selection.

## Discussion

This study, through the development of dynamic growth models for broomcorn millet and precise determination of trait thresholds, offers significant contributions to cultivation management. In agricultural practices, the identification of inflection points of plant height growth (approximately 20 days before reaching the height limit threshold) enables the establishment of a fertilization decision system for critical phenological stages: nitrogen topdressing can be applied during rapid growth phases and controlled nitrogen application during plateau phases to prevent delayed maturity and excessive vegetative growth [28]. From a genetic breeding perspective, the strong positive correlation between maximum stem diameter and lodging resistance indices [29] indicates that stem diameter thresholds derived from growth models offer a novel approach for screening stress-resistant germplasm. Furthermore, the development of chlorophyll content variation models and associated thresholds allows for the prediction of chlorophyll decline rates during heading stages. Timely foliar fertilization when values exceed these thresholds can mitigate yield losses.

Traditional phenotypic analysis methods struggle to fully capture the growth dynamics of broomcorn millet in complex environments. This study employed nonlinear methods such as the Logistic model, Richards model, and polynomial regression to fit and analyze broomcorn millet traits including plant height, stem diameter, and chlorophyll content. By constructing mathematical models that integrate plant height, stem diameter, and chlorophyll parameters, this study breaks away from traditional reliance on empirical data, parameterizes growth patterns, and advances the application of mathematics in agricultural phenotyping research, enabling precise and rapid variety selection. By fitting models using field phenotyping data, growth thresholds for different regions and varieties can be quickly obtained, providing direct evidence for cross-regional screening and directed breeding, significantly improving efficiency.

The experiment further validated the significant differences in phenotypic traits among different millet varieties through analysis of variance (ANOVA) [30]. The analysis of variance (ANOVA) for plant height showed extremely significant differences between varieties (p = 0.0000) [31]. During this period, the plant height of Pinshu 4 was the highest with outlier values, while the plant height of Jinshu 7 was the lowest with stable traits, indicating the decisive role of genetic characteristics in plant height. This provides phenotypic evidence for the targeted breeding of dwarf, lodging-resistant, or tall varieties. Stem diameter showed significant differences (p = 0.0001) after Kruskal-Wallis test. White Proso Millet 8 had the thickest stems with high data variability, while Jin Shu 5 had thinner stems with concentrated data, reflecting different lodging resistance among varieties, which can guide the selection of dense-planting varieties and planting density levels. Chlorophyll content showed significant differences after Kruskal-Wallis test (p = 0.0023) [32], with Pinshu 1 exhibiting a clear advantage in chlorophyll content, White Proso Millet 8 had lower content, reflecting the germplasm specificity of photosynthesis. These variance analysis results are consistent with the threshold characteristics of the model fit, jointly analyzing the superiority and inferiority of millet phenotypic traits among varieties, providing scientific basis for variety selection and precision cultivation in arid regions. Based on comprehensive traits, the variety ranking is as follows: Pinshu 4 > Xiaohongruan Proso Millet 6 > White Proso Millet 8 > Jinshu 7 > Huangmizi3 > Pinshu 1 > Yuanpinghongmi5 > Jinshu 5. Pinshu 4 leads due to its dual advantages in plant height and stem diameter, Xiaohongruan Proso Millet 6 follows closely with high photosynthetic efficiency, White Proso Millet 8 exhibits balanced performance in stem diameter and chlorophyll content, Jinshu 7 has stable plant height and high chlorophyll content, while the remaining varieties rank lower due to weaker performance in single or multiple traits.

Future research could further expand the model’s environmental adaptability by optimizing model parameters based on soil and climate factors in different regions. Additionally, linking phenotypic thresholds with molecular markers could enable “phenotype-gene” synergistic breeding, providing a more comprehensive theoretical foundation for the intelligent development of crop industries in various regions.

## Conclusion

This study selected eight representative broomcorn millet varieties and used Logistic, Richards, and quadratic polynomial regression models to fit the growth changes in plant height, stem diameter, and chlorophyll content for each variety. All fitted curves had extremely significant coefficients of determination (R^2^) that were close to 1, enabling the determination of the saturation thresholds and the time required to reach these thresholds for each phenotypic trait. This systematically revealed the growth dynamics differences among different millet varieties in terms of key crop phenotypic trait thresholds.The results showed significant differences among varieties in plant height (134.86–171.74 cm), stem diameter (8.47–10.28 mm), and chlorophyll content (31.17–37.40 mg/m^2^). Additionally, plant height and stem diameter exhibited asynchronous growth, with plant height reaching the threshold before stem diameter reached its maximum value. The three models showed excellent fitting performance, with R^2^ values ranging from 97.84% to 99.39% for plant height, 81.34% to 94.94% for stem diameter, and 69.48% to 87.05% for chlorophyll content, validating the applicability of the selected models to the growth dynamics of millet. The study further confirmed significant phenotypic differences among varieties using box plots, mean plots, and variance analysis (ANOVA, Kruskal-Wallis test), with p-values all less than 0.01. Pinshu 4 exhibited the best plant height and stem diameter, Xiaohongruan 6 had the highest chlorophyll content, and the chlorophyll distribution across different leaf regions was relatively uniform. Jinshu 5 showed a rapid decline in chlorophyll content, potentially indicating premature senescence. These results can provide data support for precise millet cultivation, such as adjusting fertilization based on plant height thresholds, and variety selection, such as selecting varieties with strong resistance to lodging and photosynthesis, while also filling the gap in variety-specific phenotypic threshold parameters.

Although this study has clarified the patterns of change in millet phenotypic thresholds, it has certain limitations: the experimental sites were limited, and environmental adaptability remains to be verified; most of the tested varieties originated from the North China region, with insufficient genetic diversity and representativeness; and no direct association has been established between phenotypic thresholds and crop traits such as yield and stress tolerance. Future efforts should expand experimental locations, incorporate more varieties from different regions, refine the theoretical framework for threshold application, and apply it to crop variety breeding in various regions.

## Supporting information

S1 File Raw Data of Broomcorn Millet for 2022. This compressed file contains the raw experimental data of broomcorn millet collected in 2022, covering three key phenotypic indicators across all growth periods: plant height (measured at regular intervals to reflect vertical growth dynamics), stem diameter (recorded to characterize stalk robustness), and chlorophyll content (determined to assess photosynthetic capacity). All data were acquired through standardized field measurements and stored in Excel format to support the reanalysis of phenotypic saturation thresholds and the verification of growth models in this study.
(ZIP)

## References

[1] Chen S, Hou H, Zhang X, Gao Z, Wang H, Yuan Y, et al. Relationship between nutrient accumulation in broomcorn millet (Panicum miliaceum L.) and microbial community under different salinity soils. Plant Soil. 2024;511(1–2):1285–302. doi: 10.1007/s11104-024-07046-2

[2] Kumar A, Tomar V, Kaur S. Millet genomics: bridging the gap between genome dynamics and breeding strategies. Plant Biotechnol J. 2022;20(5):803–817.35178853

[3] Dong K, Ren R, He J, Zhao Y, Wang L, Zhang Y. Breeding report of a new millet cultivar Longmi 21. Cold Arid Reg Agric Sci. 2023;2:416–9.

[4] Jones JW, Hoogenboom G, Porter CH, Boote KJ, Batchelor WD, Hunt LA, et al. The DSSAT cropping system model. Eur J Agron. 2003;18(3–4):235–65. doi: 10.1016/s1161-0301(02)00107-7

[5] Prusinkiewicz P, Lindenmayer A. The Algorithmic Beauty of Plants. New York: Springer-Verlag; 2001.

[6] Wang P, Tian H, Zhang Y, Liu J, Li C, Zhao X. Research progress on crop growth monitoring and yield estimation based on deep learning. Trans Chin Soc Agric Mach. 2022;53(02):1–14.

[7] Zeng B, Ma C, Li H, Zhang Y, Wang X, Liu J. Lightweight and simplified cultivation techniques for broomcorn millet planted after spring wheat in the Yellow River irrigation area of Ningxia. China Seed Ind. 2025;(01):156–9. doi: 10.19462/j.cnki.zgzy.20241105007

[8] Cao X, Wang J, Wang H, Li Y, Zhang Q, Liu P. Advances in the cultivation research of broomcorn millet. Anhui Agric Sci. 2015;43(31):79-81,84. doi: 10.13989/j.cnki.0517-6611.2015.31.032

[9] Li W, Sun J, Li C, Wang Y, Zhang H, Liu B, et al. Dynamic simulation and analysis of crop growth curves in a wheat-intercropped faba bean system. J Cold Arid Reg. 2025;4(01):47–52.

[10] Qiu R, Wei S, Zhang M, Liu Y, Wang H, Li J. Review of measurement methods in crop phenomics. China Agric Sci Dig Agric Eng. 2019;31(01):23-36,55. doi: 10.19518/j.cnki.cn11-2531/s.2019.0003

[11] Zhai J, Shi Z, Li J, Wang T, Liu Y, Chen B. Missing data processing method for concrete dam deformation monitoring based on GRU. People’s Pearl River. 2024;45(12):122–7.

[12] Zhang T, Wei S. Method for measurement error problems in marginal models of longitudinal data. J Zhaoqing Univ. 2015;36(02):12–6.

[13] Zhao S. Analysis and evaluation of factors affecting the fit goodness R^2^. J Northeast Univ Financ Econ. 2003;(03):56–8.

[14] Xu X, Li B. Research on the impact of different dependent variable selection on the coefficient of determination R^2^. J Taiyuan Univ Sci Technol. 2007;(05):363–5.

[15] Wu X, Zhang X. Local influence analysis and diagnosis of fit goodness in linear models. J Nankai Univ (Nat Sci Ed). 1994;(02):68–76.

[16] Xu R. Research and application of the logistic equation. J Xinzhou Teach Univ. 2011;27(05):28–30.

[17] Mo H, Chen Y. Plant growth process modeling and optimal analysis based on the logistic equation. J Jiaozuo Univ. 2006;(04):70–1. doi: 10.16214/j.cnki.cn41-1276/g4.2006.04.034

[18] Fan X, Guo G, Sun H, Li M, Zhou P, Yang Q. Experimental verification and analysis of the fractal Richards model for horizontal movement of unsaturated soil water. Environ Eng. 2019;37(12):212–7. doi: 10.13205/j.hjgc.201912037

[19] Cheng M. Parameter estimation of the Richards model and its application. Pract Recogn Math. 2010;40(12):139–43.

[20] Li Y-S, Hu D, Nie J, Huang K-H, Zhang Y-K, Zhang Y-L, et al. Genetic Analysis of Plant Height and Stem Diameter in Common Buckwheat. Acta Agron Sin. 2018;44(8):1185. doi: 10.3724/sp.j.1006.2018.01185

[21] Poorter H, Niklas KJ, Reich PB, Oleksyn J, Poot P, Mommer L. Biomass allocation to leaves, stems and roots: meta-analyses of interspecific variation and environmental control. New Phytol. 2012;193(1):30–50. doi: 10.1111/j.1469-8137.2011.03952.x 22085245

[22] Liu X, Wang R, Du H, Zhang Y, Li J, Zhao M. Morphological and anatomical differences in agronomic traits among varieties of broomcorn millet (Panicum miliaceum L.). J Shanxi Agric Univ (Nat Sci Ed). 2013;33(4):289–94. doi: 10.13842/j.cnki.issn1671-8151.2013.04.012

[23] Niklas KJ. Plant Allometry: The Scaling of Form and Process. Chicago: University of Chicago Press; 1994.

[24] Xu S. Research progress in crop growth models. Anhui Agric Sci Bull. 2023;29(04):26–32. doi: 10.16377/j.cnki.issn1007-7731.2023.04.029

[25] Li Y, Li C, Liu C, Wang H, Zhao Q, Zhou X. Analysis of QTLs associated with chlorophyll content using the G46B/A232 recombinant inbred line population. Southwest China J Agric Sci. 2018;31(11):2223–8. doi: 10.16213/j.cnki.scjas.2018.11.002

[26] Yang H, Ding Y, Cai J. Changes in chlorophyll content of functional leaves in early indica rice and its relationship with yield. Jiangsu J Agric Sci. 2022;50(03):99–104. doi: 10.15889/j.issn.1002-1302.2022.03.015

[27] Chen L, Zhang R, Zhang W, Ren X, Liu J, Zhao H. Comparison of photosynthetic characteristics and agronomic traits of different broomcorn millet varieties. Shanxi Agric Sci. 2019;47(04):553–9.

[28] Wang W, Hu B, Chu C. OsNRT1.1A: a key gene to address “green and late maturity” under high nitrogen in rice. Hereditas (Beijing). 2018;40(03):257–8.

[29] Zhao X, Zhou S-L. Research progress on traits and assessment methods of stalk lodging resistance in maize. Acta Agronom Sin. 2021;48(1):15–26. doi: 10.3724/sp.j.1006.2022.03055

[30] Feng S. An attempt to replace variance analysis with graphical method in agricultural scientific experiments. Acta Agric Boreali-Sin. 1989;(02):115–23.

[31] Zhang S, Chen Q. Application of data statistical analysis software SPSS (IV)—general factorial ANOVA (GLM). Anim Husb Vet Med. 2003;(08):24–6.

[32] Pu H. Introduction to the principle of Kruskal-Wallis test and its application. J Xingyi Norm Univ Natl. 2019;(04):108–11.

